# COVID-19 masks and limited number of shoppers as determinants of shop assistants’ (dis)honesty

**DOI:** 10.1371/journal.pone.0296746

**Published:** 2024-01-11

**Authors:** Maciej Koscielniak, Dorota Marciniak, Dariusz Doliński

**Affiliations:** 1 Institute of Psychology, SWPS University of Social Sciences and Humanities, Warszawa, Poland; 2 Interdisciplinary Center for Applied Cognitive Studies, Warszawa, Poland; 3 Center for Social Behavior Research, Wroclaw, Poland; Universite Paris Pantheon-Assas, FRANCE

## Abstract

Previous laboratory and field studies have demonstrated that the dishonesty of commercial transaction participants may depend on subtle cues. In this field study conducted on a sample of 216 shop assistants in Poland, we planned to demonstrate that coronavirus disease-related factors could result in an increased propensity for dishonesty among shop assistants. This investigation is unique in its application of social psychological theories to illuminate hitherto unexplored side effects of combating the coronavirus disease 2019 pandemic. Our supposition was that the potential detriment encountered by individuals wearing solid surgical masks would involve being viewed as more abstract and remote, thereby heightening the likelihood of being deceived by a vendor. Moreover, we examined the potential relationship between the limited number of customers in retail establishments (related to pandemic restrictions) and the unscrupulous practices of sellers—specifically the act of retaining change. The effect of wearing masks was statistically non-significant, whereas the impact of other customers’ absence was significant. Moreover, unexpected results related to transaction parties’ genders were obtained, showing that shop assistants tended to be more honest when dealing with customers of the same gender. The results are discussed in the context of empathy toward masked customers, self-awareness theory, social norms of honesty, and identification with gender groups.

## Introduction

Although civic honesty is known to be an important determinant of economic welfare in societies based on cooperation [1], people do not always behave morally. Over four decades ago, Robert E. Wilkes estimated $2 billion as the cost of annual shopping fraud prevention in the US [2]. Wilkes focused on consumer-initiated fraud in the marketplace, a problem that has been investigated in numerous studies [3–5]. However, in the present study, we focused on the opposite party in shopping transactions. We intended to demonstrate that the seller (shop assistant) may also be tempted to make extra money through simple fraudulent acts (for example, not returning excess change). While previous studies have examined this phenomenon [1,6,7], ours is the first of its kind in the context of the coronavirus disease 2019 (COVID-19) pandemic.

Like most countries worldwide, Poland introduced several restrictions to manage the spread of COVID-19 between 2020 and 2023. These included wearing masks, banning large gatherings, and—at a certain point—even stopping people from leaving their homes without a compelling reason. By the time we began our study in the summer of 2021, most of these rigid restrictions had eased. However, it was still obligatory for people to wear masks inside stores and other indoor common spaces. While public concerns and burdens related to COVID-19 restrictions have been subjects of prior empirical analysis [8], we uniquely contextualize these challenges within the framework of social psychology. The ongoing restrictions along with the former rule limiting the number of people allowed in indoor spaces (one person per 10–20 square meters) served as the catalyst for this study [9]. We hypothesized that social distancing and face masking in retail spaces exert a psychological impact on ethical behavior, potentially reducing people’s susceptibility to social control or influence.

### Psychological underpinnings of dishonesty

Drawing on psychological theories and empirical evidence from preceding studies on general dishonesty, we sought to examine the previously unknown side effects of COVID-19. We aimed to provide fresh insights into the social and political ways of dealing with the pandemic. Our goal was to demonstrate that reducing social interactions by covering faces and maintaining distance (often allowing only one customer at a time in smaller shops) carries several negative side effects. Specifically, these measures can inadvertently loosen social norms and lead to tangible material losses.

It has been demonstrated that anonymity-related darkness can be a strong cue toward dishonest behavior [10]. Specifically, immoral actors feel less threatened by punishment when they perceive the environment as anonymous and safe. This effect was also confirmed by Zimbardo’s study [11], in which participants wearing camouflage hoods (making them difficult to recognize) agreed to engage in cruel and immoral acts toward others. We believe that this can also be the case for surgical masks used because of COVID-19. Masks are a confirmed measure of effective protection against the propagation of the virus [12]; simultaneously, covered faces lead to a quality change in human interaction. Faces are the most important sources of inference when meeting new people [13]; faces enable basic social processes for recognition [14]. The lack of such possibilities while encountering an anonymous individual can eliminate constraints related to social ethics, resulting in dishonesty or aggression in both the real [15,16] and virtual worlds [17].

While most of the abovementioned studies were concerned with masked/anonymous actors in social interactions, it seems apparent that an observer’s mask-wearing could also result in the seller’s increased propensity for dishonesty. This relationship was confirmed in both laboratory and natural environments by Ahmed [18], who referred to Milgram’s theory when discussing his results. Specifically, he suggested that people react more unethically toward remote and invisible victims for five basic reasons: lack of empathic cues, narrowing of the cognitive field, the phenomenal unity of the act, incipient group formation, and acquired behavioral disposition [19]. We posit that the example of the bombardier from Milgram’s paper is particularly relevant here. Although the bombardier is aware of the deadly consequences of his actions, the invisibility of the victims strips his knowledge of any emotional impact. Therefore, in the present study, we intend to investigate whether masks worn by shoppers lead to greater dishonesty from retailers—because of inducing remoteness and a reduction in empathy within mutual seller–customer interactions.

Considering the aforementioned studies, it seems plausible that the mere presence of other people (in addition to the interacting parties) should decrease sellers’ propensity for dishonesty. Other people may be perceived by dishonest actors as possible witnesses to their behavior, thereby threatening their sense of anonymity while committing unethical acts. According to the theory of objective self-awareness [20], transgressing social and moral norms may be more difficult owing to a perceived awareness of being observed and evaluated by others. This social context factor is strongly underlined in a systematic review by Guerin, who concluded that humans naturally tend to seek social approval, which results in conforming to social norms in the mere presence of others [21].

Apart from the effects of COVID-19 masks and the presence of others, we expected that the demographic factors of customers’ age and gender would modify shop assistants’ propensity to cheat. Namely, we expected that younger (teenage) customers and women would be less endangered by sellers’ malicious actions, in accordance with the norm of social responsibility, whereby women, children, older adults, or those with disabilities are usually seen as more vulnerable [22]. Although this norm is particularly pronounced in relation to children, it also elicits protective attitudes toward teenagers—whose consumer socialization is not fully developed [23]. Overrated beliefs in gender-role stereotypes often lead to caring and altruistic behaviors toward those who are perceived as weaker and dependent on others [24].

Moreover, the gender of the shop assistant is expected to be significantly related to dishonest behavior. In a previous field study, women were more likely to return excess change in restaurants [5], which, as Tibbetts [25] suggests, could arise from women’s stronger tendency to feel the dishonesty-related emotion of shame compared with men. We also controlled for seller–customer gender compatibility, hypothesizing that, along with the gender solidarity hypothesis [26,27], shop assistants would be less willing to cheat when perceiving the buyer as an in-group member. It is indubitable that gender is one of the most situationally accessible social categorizations [28] used to recall information and make judgments about others [29].

When proposing the aforementioned demographically based hypotheses, we are aware of their partial contradictions. This problem is mainly gender-related: on the one hand, male vendors should be more honest toward women (stereotypically perceived as more vulnerable customers); on the other hand, they should react more honestly toward men (members of their own social group). However, considering the strong empirical evidence, we believe that, despite the described contradiction, it is reasonable to make such competing hypotheses.

Altogether, we aimed to investigate the consumer risk of being a victim of dishonesty while shopping in grocery stores. COVID-19-related changes (wearing surgical masks, reducing the number of shoppers owing to regulations, and social distancing) were expected to impact shop assistants’ propensity to cheat by keeping the excess change received. These factors were investigated together with demographic variables such as customers’ age category and gender, and sellers’ gender.

Considering future epidemic threats, finding answers to our research questions is essential for planning social policies. Scholarly conversation often overlooks the less obvious but significant consequences of pandemic restrictions, such as increased feelings of anonymity or social isolation. This study aimed to highlight that these effects come with their own costs. They are not limited to economic or material values, but are importantly tied to the weakening of interpersonal relationships and erosion of social trust. Noticing these costs is crucial for developing informed policies that will protect social fabric during times of crisis.

## Materials and methods

### Research ethics statement

All the procedures were conducted in accordance with the ethical guidelines of the American Psychological Association. No deceptions were included in this study; only retailers’ routine daily behavior was observed. The study was approved by the Ethics Committee of SWPS University of Social Sciences and Humanities, Faculty of Psychology in Wroclaw (consent number 03/P/01/2022). Given the nature of the field study, informed consent was not obtained.

### Preparation

Before beginning the study, a complete list of grocery shops belonging to one retail chain (shops located in Poznan, PL, and nearby towns) was prepared. Using a random number generator, the shops were assigned to one of four study research assistants (responsible for carrying out the procedure) and one of two experimental conditions (surgical mask vs. transparent visor). In the randomization preferences, it was established that every research assistant should make the same number of visits for both types of face covering. A map of all the shops visited by the research assistants, along with the mask condition assigned to each store, can be found at the OSF project’s website (https://osf.io/e4afb).

### Participants

The study was conducted with shop assistants in 216 grocery shops. The majority of the participants were women (78.24%). All shop assistants used surgical or textile masks to cover their faces, according to legal regulations in Poland during this period. Salespersons were not informed about their participation in the procedure either before or after (according to APA ethical guidelines related to situations when debriefing procedures could result in moral harm, https://www.apa.org/ethics/code 8.08b). Not obtaining informed consent in field experiments is typically recommended because the awareness of such participation can engender non-authentic behaviors, thus contaminating the data with social desirability bias [30].

### Procedure

The research assistants (shoppers) were two adults aged approximately 50 years (man and woman) and two teenagers (18 years old, man and woman). While entering the shop, the research assistant wore one of two types of protective masks on their face, according to the experimental condition to which the shop was assigned. In half of the cases, this was a surgical mask (non-transparent, covering the nose and the entire lower half of the face), whereas in the other half, a plastic visor was used (revealing the entire face).

All shops selected for participation in the study were visited at a similar time, between 10 am and 4 pm (previous dishonesty research indicates that the time of day can impact the honesty of participants [31]). The research assistant entered the shop designated by the random draw and verified three prerequisites for conducting the procedure: (1) no more than three customers could be present in the store; (2) only one salesperson was allowed to be present; and (3) the availability of a specific brand of ice cream (called “Cactus”) in the freezer. If any of these prerequisites was not fulfilled, the research assistant would immediately leave the shop (this particular store could be visited once again, but not earlier than the following day, and always by another research assistant). Additionally, if a vendor engaged in any form of unexpected interaction with the research assistant or others (becoming involved in a dialogue, helping another customer, another vendor unexpectedly appearing, etc.), it would be considered a contamination of the experimental procedure, resulting in the exclusion of this shop from further analysis. In summary, of the 281 shops visited during the study, 65 were not included in the data analysis: approximately two-thirds owing to the unavailability of Cactus ice cream, and the remaining one-third owing to the contamination of the research procedure. All vendors participating in the study wore medical (non-transparent) masks, which was a mandatory legal requirement at the time of the experiment.

When all the inclusion criteria were met, the research assistant approached the freezer, took a single Cactus ice cream, and promptly approached the counter. The price of this ice cream was always the same, set at 2.30 PLN (according to the chain’s price list). The research assistant put 3.30 PL (coins: 3 x 1 PLN, 20 GR, and 10 GR) on the counter, explicitly pronouncing: “Exact change!” (Letting the seller know that the given money was intended to be the exact amount to be paid.) Then, the research assistant slowly walked away from the counter, ensuring that the seller counted the money received (no counting occurred in three shops, which resulted in their exclusion from the database).

The main dependent variable in the study was the returning (or not) of the overpaid change by the shop assistant, who, upon discovering the overpayment, could either address the customer and return the excess money or stay silent about it. This variable was recorded on the survey sheets as soon as the research assistant left the shop (along with other variables, such as the presence of other people in the shop and the gender of the shop assistant).

The number of shops visited by the research assistants wearing the transparent visor versus surgical mask was the same (108 in every condition), and equal proportions also applied to the age (teenagers vs. adults) and gender (women vs. men) of research assistants. There were more shops with the research assistant as the only customer (n = 122) compared with those with bystanders (n = 94), but the difference was not statistically significant; x^2^ (1, N = 216) = 3.63, p = .057. Finally, there was a significant difference in the gender of shop assistants (47 men vs. 169 women); x^2^ (1, N = 216) = 68.91, p < .001.

The study lasted approximately 10 weeks during the summer months (June–September) of 2021. The authors had no access to information that could identify individual participants (vendors) during or after data collection: the addresses of the visited shops were recorded in a separate database, making it impossible to pair them with the actual data collected in the experiment.

## Results

SPSS Statistics for Windows, version 27 (IBM Corp., Armonk, NY, USA) was used to analyze the data. As the dependent variable could only occur at two levels (0 = *no excess change returned*, 1 = *money returned*), logistic regression was chosen as the analytical method (for further information, refer to “Materials and methods”). To verify the hypotheses, two main predictors were entered: type of COVID-19 mask (0 = *transparent visor*, 1 = *surgical mask*) and the presence of other customers in the shop (0 = *no other customer present*, 1 = *others present*). Several additional predictors were also entered to control the demographic variables: gender of the research assistants and sellers (0 = *men*, 1 = *women*), gender compatibility between research assistant and seller (0 = *false*, 1 = *true*), and age of the research assistants (0 = *teenager*, 1 = *adult*). All the entered predictors were dichotomous.

The regression model was statistically significant, x^2^ (6, N = 216) = 30.54, p < .001. It explained 17.6% (Nagelkerke *R*^2^) of the variance in returning excess change by shop assistants, with a sensitivity of .53 and specificity of .74. Thus, the hypotheses were partially confirmed. Although the direction of the COVID-19 mask effect was in line with our expectations, and research assistants wearing transparent visors were more likely to be refunded (by 21% compared with those wearing surgical masks; odds ratio (OR) = .79, 95% confidence interval (CI) [.44, 1.41]), the coefficient of this effect was statistically non-significant. By contrast, the effect of the presence of other customers was fully confirmed; shopping in the presence of other customers made the odds of being honestly treated over three times higher than was the case with those who were alone with the shop assistant (OR = 3.44, 95% CI [1.90, 6.20]).

**Fig 1:**
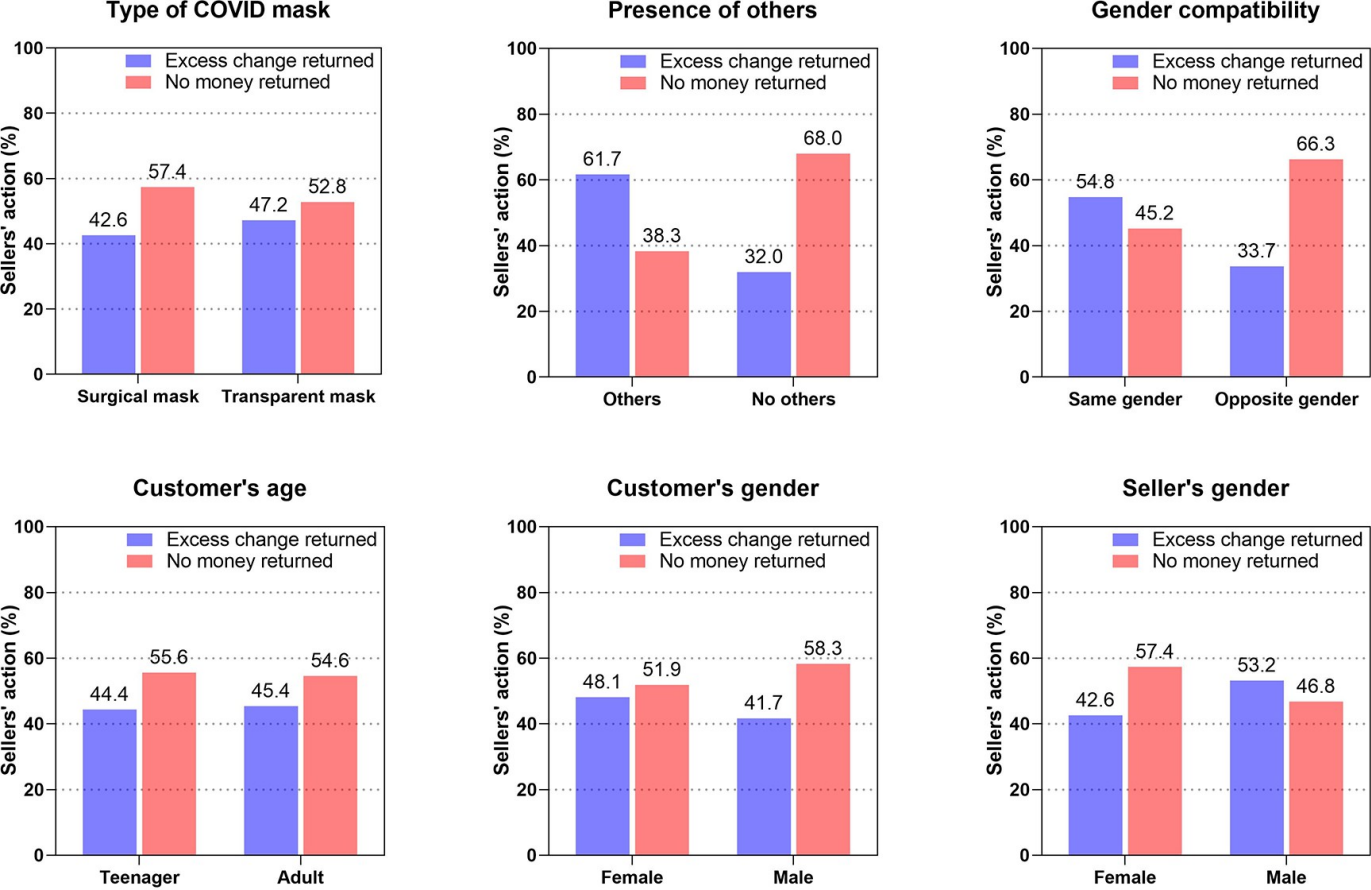
Sellers’ Honesty. Bar plots depicting the relationship between all variables manipulated and controlled for in the study and the frequency of sellers’ honest/dishonest behavior.

Among the demographic predictors, only gender compatibility between the research assistant and shop assistant turned out to be a significant predictor of the odds of receiving excess change. Along with our expectations based on the gender identity hypothesis, the odds of receiving the correct amount of change were much higher for research assistants of the same gender as the sellers (OR = 2.84, 95% CI [1.38, 5.84]). None of the other demographic variables (research assistant’s gender and age and seller’s gender) were significant predictors of shop-floor dishonesty among sellers. The frequencies of the sellers’ actions are presented in Fig 1, and the complete set of regression coefficients is listed in Table 1.

**Table 1:** Logistic regression analysis for the association between the seller returning excess change and examined variables.

	B	SE	z	OR	95% CI	
					lower	upper
**(Intercept)**	-1.56	.45	-3.45	.21		
**Mask (Surgical)**	-.24	.30	-.80	.79	.44	1.41
**Presence of others (True)**	1.23	.30	4.10	3.44	1.90	6.20
**Gender compatibility (True)**	1.04	.37	2.83	2.84	1.38	5.84
**Customer’s age (Adult)**	.26	.30	.86	1.30	.72	2.35
**Customer’s gender (Man)**	.32	.37	.88	1.38	.68	2.83
**Seller’s gender (Man)**	.31	.37	.85	1.36	.66	2.80

Note: B = logistic regression estimate; SE = standard error; OR = odds ratio; 95% CI = odds ratio confidence intervals (lower and upper).

## Discussion

In concordance with our research hypotheses, the main aim of this project was to verify the relationship between shop assistants’ honesty/dishonesty and contextual (COVID-19-related) factors. First, it was expected that wearing surgical masks (compared with transparent visors) would limit the propensity of shop assistants to return excess change. Second, the mere presence of other customers would be linked to more ethical decisions by sellers. Finally, the demographic variables related to the seller–customer interplay were examined (gender of both actors, compatibility of genders, and customer age). The results of the statistical analyses allowed for a partial confirmation of the hypotheses; shop assistants were confirmed to act more honestly (1) in the presence of bystanders and (2) when serving customers of similar gender to their own. All other hypothesized effects were statistically non-significant.

Particularly perplexing was the absence of substantial support for our primary research hypothesis, which postulated differences in honesty levels toward customers wearing surgical masks compared with those donning transparent visors. Despite our hypothesis being robustly anchored in the extant literature and theoretical frameworks outlined in the introduction [18,19], the anticipated effect emerged as too faint to attain statistical significance. This outcome may be attributable to the possibility that the masking effect—referring to the sense of anonymity [10,11]—exerts a more potent influence when it involves the perpetrator of dishonest behavior (the seller) rather than the victim. However, we advise caution in interpreting this result. The lack of statistical significance does not equate to the non-existence of the effect, but rather serves as a stimulus for further investigation, potentially involving a larger participant sample.

The strongest statistical effect, increasing the odds of a salesperson’s honesty by over 243%, was observed in the case of the presence of bystanders (other customers). The environment in which the actor is accompanied by other people has been examined in multiple studies, providing different results in various contexts. According to the deindividuation hypothesis [32], the presence of others should result in the loss of individual ethical norms, resulting in behaviors that are typically inhibited, as individuals feel less accountable for their actions. Conversely, according to the social facilitation theory [33], the presence of an audience normally increases the tendency to perform most routine and default actions—enabling individuals to perform everyday tasks more efficiently under observation [34]. Alongside this second theory, our results optimistically suggest that the sellers’ default response is honesty, while misconduct results from reflection and a deliberative desire to maximize expected payoffs when no witnesses are present. Honesty also seems to be significantly related to self-monitoring and self-awareness, typically intensified in the presence of bystanders (as has been confirmed in previous studies [35]). Finally, the possible activation of social norms of honesty by the mere presence of others must be emphasized. As Cialdini and Trost wrote, “Social norms are rules and standards that are understood by members of a group, and that guide and/or constrain social behavior without the force of laws” [36], which could be perfectly observed in our study.

The significant effect of gender compatibility between customers and sellers indicates a noticeable niche in scientific research. Although identifying oneself as a man or woman is a salient social categorization cue [37], there has been little scientific interest in studies on gender solidarity [26,29]. In accordance with numerous sociopsychological theories such as the social identity theory [38], unethical behavior toward in-group members is less likely compared with deceiving members of the out-group [39]. Our results were also important considering the aforementioned competition in demographic hypotheses: the gender solidarity effect seemed to be stronger than the rule of traditional kindness and courteousness toward women, which did not turn out to be significant. However, further investigation of this topic is strongly recommended in retail contexts as well as in generalized dishonesty research.

### Practical implications

Our study provides several practical insights important for policymakers responsible for establishing regulations during a health crisis, such as the COVID-19 pandemic. First, it appears that the act of a customer wearing a mask does not significantly affect the probability of retail fraud. This outcome needs additional confirmation, particularly when considering the mask-wearing actor (perpetrator of fraudulent behavior) [10,11] possibly being more influenced by the illusion of anonymity and impunity.

Second, we confirmed that the lack of other customers or bystanders in stores might induce more dishonest behavior from salespeople. While maintaining social distance is vital for public health [12], such policies could unintentionally prompt fraudulent business transactions. Therefore, retail outlets should consider establishing more rigorous monitoring systems when customer presence is low, and customers should be more vigilant to minimize the risk of being cheated.

Lastly, a concerning outcome of our study was the identification of unethical gender solidarity, characterized by retailers’ increased propensity to engage in deceptive practices toward customers of the opposite gender. Although this finding is not directly related to the pandemic, it presents a compelling dynamic when viewed against the backdrop of two contradictory psychological tendencies: the propensity for same-sex support and identification [26] contrasted with intrasexual competition tendencies, which involve more competitive behavior toward the same sex and increased prosocial attitudes toward the opposite sex [40]. In this study, gender solidarity between the vendor and customer emerged as the predominant tendency, warranting further investigation to determine if this represents a general phenomenon. Furthermore, our results highlight a previously unexplored issue in the shopping environment, namely possible discrimination against customers of the opposite sex. This finding underscores the need for targeted educational interventions for salespeople, aimed at promoting fairness and respect toward all customers, thereby cultivating a more equitable and respectful shopping atmosphere.

### Limitations

This study had some limitations. We made great efforts to ensure a balanced number of observations in all aspects of the study (men vs. women as research assistants, transparent vs. surgical masks, presence of other people vs. solitary purchases); however, this proportion could not be maintained with respect to the gender of salespeople, who were mostly (78.24%) women. Notably, although the group of male salespeople was substantial (47 individuals), the observed results were particularly applicable to predicting the behavior of saleswomen, and additional research on men would be worthwhile.

Shopping and selling in grocery stores usually involve automatic processing rather than deliberation, low sums of money being exchanged, and fast transaction times [41]. Consistent with the results from previous studies, this automatic/intuitive way of processing information can be linked to a higher propensity for dishonesty (as not associated with “real” social harm [42] and not resulting in serious self-maintenance threat [43]). Therefore, it would be interesting to replicate these results in a more deliberative consumer environment to be able to generalize them to a wider scope of economic transactions.

We acknowledge that, in addition to the limitations already cited, significant constraints of our survey stemmed from both the methodology of data collection and the sample size. Employing real-life shopping scenarios as our experimental setting restricted our ability to manipulate the masking of the salesperson’s face. We were only able to modify the consumers’ facial covering, which possibly resulted in a less pronounced effect of anonymity in the shopping interactions. Furthermore, a notable limitation pertained to the number of stores belonging to a particular retail chain within and surrounding the city. This factor constrained our ability to conduct the study across a more extensive number of shopping situations.

Finally, it is important to acknowledge that the study was not executed under a double-blind design. The assistants were aware of the research hypotheses, which may have potentially influenced their conduct within the experimental context.

### Conclusion

Our study makes significant contributions to extant theoretical frameworks by expanding upon the social facilitation theory and underscoring the role of social norms in shaping behavior. The findings support the idea that an audience increases the likelihood of honest behavior among salespersons; this what bolsters the understanding of honesty as a behavior influenced by social context.

We uncovered the relatively unexplored role of gender compatibility in influencing salespersons’ honesty. The salespersons were less likely to deceive customers of the same gender, lending support to theories such as the social identity theory. The gender solidarity effect supersedes traditional norms of courtesy toward women, highlighting the complexity of dynamics in retail settings and emphasizing the need for further investigation of gender effects in dishonest behavior research.

In conclusion, despite its limitations, our study provides intriguing findings concerning any individual doing their daily shopping during the pandemic. We failed to confirm a significant effect of consumers’ mask-wearing on the likelihood of their exposure to seller dishonesty (although it is possible that this effect would be stronger for sellers’ mask usage). However, we found novel evidence for the importance of the social context of shopping, related to the presence of other people as unintentional witnesses to transactions. Referring to the psychological debate on the default preference for honesty or dishonesty [44], our results support the hypothesis of the default honesty of sellers, which can be further enhanced by social and demographic cues such as the mere presence of witnesses to the transaction and the gender solidarity of traders. This first finding seems particularly relevant to the COVID-19 restrictions in most countries, which regulate the maximum number of people simultaneously present in commercial spaces. Increased retail dishonesty related to the limited number of bystanders can be considered a side effect of the COVID-19 pandemic, which is commonly ignored but possibly affects everyone.
